# Blasting ore size detection based on efficient dehazing network and multi-dimensional feature fusion

**DOI:** 10.1038/s41598-026-39514-3

**Published:** 2026-02-28

**Authors:** Pingfeng Li, Shoudong Xie, Wanzhong Zhang, Deming Chen, Sheng Peng, Weibin Fang, Dehao Zeng

**Affiliations:** 1 Key Laboratory of Safety Intelligent Mining in Non-Coal Open-Pit Mines, National Mine Safety Administration, Zhaoqing, 526530 China; 2 Hongda Blasting Engineering Group Co., Ltd., Changsha, 410011 China; 3 Dongguan Chinese Sciences Cloud Computing Academy, Dongguan, 523808 China; 4 College of Information Science and Engineering, University of Jinan, Jinan, 250022 China

**Keywords:** Particle size of ore, Deep learning, Object detection, Efficient network, Dust removal network, Multi-scale feature fusion, Engineering, Mathematics and computing

## Abstract

Ore particle size distribution is an important metric for evaluating blasting outcomes and affects the energy consumption of ore crushing equipment. Faced with dense accumulation of ore, nonuniform size distributions, dust occlusion, and target loss due to motion, using computer vision methods, we propose a blasting ore size detection method based on efficient dehazing network and multi-dimensional feature fusion, which is an improvement to YOLOv8. Firstly, we constructs an efficient defogging backbone network that combines feature attention and composite scalable backbone so that the model can efficiently extract the features of ore images and enhance the robustness of the model to dust interference in the ore crushing process. Secondly, we introduces a new feature fusion network that combines the convolution model and the Vmamba sequence model as well as cross-layer fusion of multi-scale features so that the model can effectively adapt to the dramatic scale change of blasting ore, capture fine ore and large-size ore, avoid ore omission, and improve the accuracy of particle size statistics. Finally, the multi-dimensional feature fusion ability of Dynamic Head was introduced to optimize the target detection head, and the feature fusion was further optimized so that the feature tensor obtained from the ore image was adapted to the detection and positioning task of ore, and the discrimination ability of the model for ore was improved. Experiments were conducted on a manually labeled jaw fracture ore dataset. Compared to the YOLOv8n algorithm, the average precision ($\overline{P}$) for detecting eight size categories of ore increased by 7%. On datasets containing interference such as smoke, dust, and wet conditions, the mean average precision at the IoU threshold of 0.5 (mAP50) improved by 7.6%. For fine ores below D5 (72 mm), the detection precision ($\overline{P}$) increased by 18.8%, while the recall rate ($\overline{R}$) rose by 13.8%. On the total one-class dataset, the recall rate ($\overline{R}$) and mAP50 reached 84% and 88.1%, respectively.

## Introduction

Ore particle size serves as a critical parameter for evaluating the effectiveness of ore blasting and influences the subsequent energy consumption in ore crushing. The accurate acquisition of ore particle size information facilitates the quantification of blasting outcomes, enhances the design of blasting schemes, and ultimately contributes to improved energy efficiency. Traditional ore size detection mainly relies on manual screening^1^ and settlement methods^2^. Although these methods are simple in principle, they have the problems of slow detection speed, poor real-time performance, and insufficient accuracy and are easily interfered with by human factors. For example, equipment such as a vibration screen will change the particle size characteristics of ore because of mechanical action and cannot accurately reflect the actual particle size distribution. With the rapid development of image processing technology and machine vision, the ore size detection based on image processing has replaced the manual method. The traditional algorithm uses the conventional image processing technology to analyze the ore particle size, including gray-scale, filter denoising, edge detection (Canny)^3^, watershed segmentation^4^, and particle size statistics^5^. Through the conversion relationship between pixel area and actual particle size, the ore particle size distribution information is obtained, and the non-contact detection of ore particle size is realized. Zhang Jianli et al.^6^ proposed an ore image denoising method constructed by lifting wavelet and an improved watershed algorithm for ore image segmentation. This kind of method is simple and low-cost and suitable for static laboratory samples. However, it handles ore adhesion poorly, and watershed segmentation easily leads to over-segmentation; the detection results are also sensitive to illumination changes and dust.

In recent years, the application of deep learning in ore particle size detection has gradually increased, mainly in the field of target detection and image segmentation. In the field of image segmentation, Liu et al.^7^ proposed an ore contour detection algorithm based on Res-Unet and U-Net networks to solve the problem of ore shadow adhesion, which improved the detection effect. Gu Qinghua et al.^8^ proposed an image segmentation method to improve the stacking and adhesion problems of broken ore by using residual deforming convolution and cavity convolution to improve the HED (Holned-nested Edge Detection) network model. Li et al.^9^ proposed a DDR-Unet (Deformable Dense Residual U-Net) algorithm, which improved the codec and other modules of classic U-Net to improve the segmentation performance under the conditions of ore covering and adhesion. The image segmentation method can effectively obtain the exact shape and edge of the ore, but there are still some limitations in the detection of blasting ore particle size. The segmentation algorithm usually has high computational complexity and poor real-time performance, and the segmentation results contain accurate edge information. However, for large-scale real-time ore particle size identification tasks in the minefield, only the length and width of the ore need to be obtained, and the accurate edge contour contains a lot of redundant information, resulting in a waste of computing power. Moreover, it is difficult to obtain the segmentation data of ore, which requires a lot of manpower and is difficult to obtain large-scale segmentation data.

In the field of detection, Bo Jingwen et al.^10^ introduced Mobilenetv2^11^, with space and channel dual attention mechanisms to improve the YOLOv3 network, which is more suitable for dispersed ore targets than accumulation targets. Gao Xinyu et al.^12^ introduced a feature pyramid^13^ and fused feature maps of different sizes of three scales to build a deep target detection network based on a convolutional neural network for dynamic identification of coal gangue particles. Xie Tao^14^ introduced the attention mechanism in the Faster R-CNN network to detect various particles in the ore components and achieved better detection results. Xie Baojia^15^ proposed an EfficientDet-X target detection model to solve the problems of small and dense pellets and irregular sinter contours. This fuses channel attention and dual attention in the backbone network and the feature fusion network and optimizes the convolution mode of the feature fusion network and the prediction network, which yields good detection effects for the two kinds of ores. The above detection algorithm has a good detection effect for ore with uniform size in general scenes and is optimized for the characteristics of the studied ore, but it still has certain limitations for mine blasting ore detection.

This paper studies the ore after blasting, and the scene is the ore at the feeding port of the jaw crusher and the conveyor belt. After blasting, the ore has a great scale change, including fine ore below 50 mm and large-sized ore above 500 mm. In the process of conveying to the feed port, a lot of smoke and dust can be produced, which has a huge impact on the detection effect of the detector. To suppress the dust, the ore is usually wet by sprinkling water, but this will change the color and visual texture of the ore so that the ore is integrated with the surrounding ore, and the densely packed ore becomes more difficult to distinguish. These contradictions make it difficult to detect ore particle size based on vision. Because of these difficulties in blasting ore size detection, we propose a blasting ore size detection algorithm based on an efficient dust removal network and multi-dimensional feature fusion.

Existing approaches to blasting-ore size estimation span a spectrum from classical image-processing pipelines (thresholding, edge detection and watershed) and 3D/depth-based reconstruction (stereo, structured light, LiDAR) to modern deep-learning detectors and instance-segmentation frameworks. Traditional 2D image-processing methods are simple and low-cost but are highly sensitive to illumination, dust and target adhesion and therefore perform poorly in realistic blasting environments; 3D solutions provide more direct metric measurements but incur high hardware, calibration and maintenance costs and are often impractical for continuous on-site monitoring. Pure detection networks (e.g., one- and two-stage object detectors) offer favorable runtime and mature tool-chains but tend to underperform on dense, small and occluded fragments unless coupled with task-specific enhancements; instance-segmentation methods can better separate touching fragments but at the expense of heavier annotation and higher inference cost. Recent transformer-style or global attention necks bring powerful contextual modeling but their quadratic memory/time complexity limits applicability on high-resolution industrial imagery. In contrast, our approach targets a middle ground that prioritizes robustness and deployability: an end-to-end trainable dehazing backbone (FFA-Net) to recover high-frequency detail under dust, an I3SS neck that fuses multi-kernel local features with linear-cost sequence modeling (Vmamba/SS2D) to capture long-range context for dense stacks, and a Dynamic Head to adaptively weight scale/task/spatial cues. This integrated design retains the implementation and runtime advantages of 2D detector pipelines while substantially improving resistance to dust, small-object detection and de-adhesion—offering a pragmatic trade-off between the high precision but costly 3D methods and the high-throughput but less robust vanilla 2D detectors.

The main contributions of this paper are as follows:

1. A dust-resistant end-to-end framework for mineral size detection was proposed, in which the fog removal module based on FFA-Net is jointly optimized with the detection network. Different from the traditional method that treats image enhancement as an independent preprocessing step, our approach enables the gradients to propagate through the fog removal network, thereby achieving feature enhancement for the mineral detection task;
2. A novel I3SS feature fusion module was introduced at the neck of the detector, which combines multi-scale convolution operations with sequence modeling based on Vmamba. This design is different from the existing fusion strategies that only use convolution or only use transformers, as it can simultaneously capture local texture details and long-range spatial dependencies. This is particularly effective for dense-packed ore with significant scale variations;
3. An adaptive detection head based on dynamic heads is introduced to further enhance the robustness in complex industrial conditions. Compared with the fixed head design in the standard YOLOv8, this module can dynamically adjust the weights of spatial, scale, and task-specific features, thereby reducing sensitivity to dust, motion blur, and partial occlusion.
4. A dataset for detecting crushed ore was constructed, which included various environmental and ore conditions and was classified by ore particle size. The effectiveness of the proposed method was verified on this dataset.

Overall, these contributions provide a practical and implementable solution for estimating the size of ore particles. This solution achieves a balance in terms of accuracy, resistance to environmental interference, and operational costs—unlike existing methods, it optimizes through joint enhancement and detection, introduces sequence-aware multi-scale fusion, and implements reasoning mechanisms that are suitable for on-site deployment.

## Methods

### Detection model construction

In order to realize the industrial deployment requirements of the ore granularity detection system in the actual mine environment, we introduce the YOLO family of algorithms. YOLO is one of the current mainstream one-stage object detection frameworks, which has the advantages of lightweight structure, fast inference speed, and high detection accuracy, and is widely used in the field of industrial vision. The YOLO series algorithm is a rapidly updated algorithm, and YOLOv8 ~ YOLO12 is the newer YOLO algorithm in recent years. Through actual tests, YOLOv8 ~ YOLO12 has similar detection performance indicators in our scene. Among them, YOLOv8 and YOLO11 are provided by Ultralytics with complete open source software frameworks, other YOLO algorithms are proposed by research institutions or universities, and the maturity of the framework is weaker than that of Ultralytics. YOLO11 is still under frequent update, and YOLOv8 is relatively stable. To improve performance indicators, model modifications often lead to increased consumption of storage and computing resources. Compared with the larger YOLOv8 variants, YOLOv8n has significantly lower parameter count, computational load, and inference latency. YOLOv8n has much greater room for improvement. Therefore, we choose YOLOv8 as the baseline detection model in this study. YOLOv8 introduces the design of decoupling detection head, RepConv structure and anchor adaptive mechanism to improve the detection performance of multi-scale targets. In addition, YOLOv8 has good scalability, visualization tool support and deployment adaptation capabilities, which greatly simplifies the training and online process of the model, and meets the application requirements of “landing, maintainability and real-time operation” of the ore size detection system.

Although YOLOv8 is a strong baseline with both accuracy and efficiency, it still has several limitations in blastmine detection tasks: It is easy to lead to performance degradation under extreme conditions, such as dense dust, motion blur, or severe occlusion, which are common in mining sites. The model exhibits sensitivity to small ore fragments, where fine-grained detection remains challenging. While efficient, YOLOv8’s feature fusion design is still convolution-centric, which limits its ability to capture long-range dependencies compared to new transformer-based methods. The architecture is mainly optimized for general object detection tasks and not for domain-specific industrial applications, which means that its adaptation to blast-ore images is insufficient without our proposed modifications. Therefore, the blasting ore particle size detection model proposed in this paper is constructed based on the target detection algorithm of YOLOv8, and the network structure is designed and improved according to the characteristics and difficulties of blasting ore detection.

**Fig. 1:**
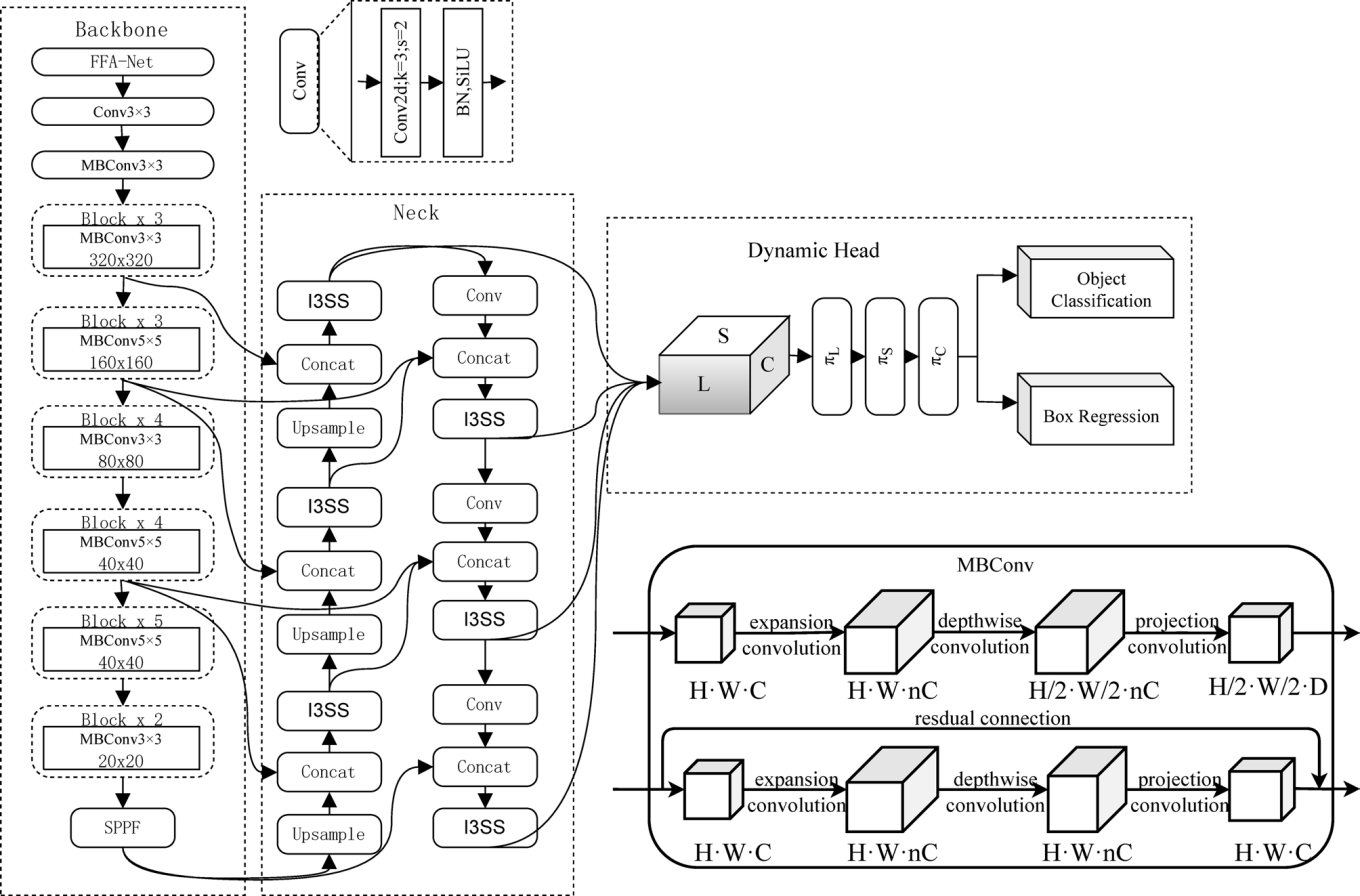
The overall network structure of the blasting ore particle size detection model.

The network architecture of the algorithm is shown in Fig. 1, and the model consists of three parts: the efficient dust removal backbone based on FFA-Net and MBConv, the feature fusion neck based on I3SS and cross-path fusion, and the multi-dimensional detection head based on Dynamic Head are used to improve the detection accuracy and robustness of ore in complex environments. The connection relationship between each module in Fig. 1 is shown by the arrow, and the corresponding pseudocode of the overall algorithm is shown in Algorithm 1. After the image is input into the network, it is firstly sent to FFA-Net for dust removal feature enhancement, then the features are extracted through the MBConv backbone, and then, the features are fused through the neck network. The fused feature map enters the detection head to output the prediction box classification, position, and boundary, and finally, the ore size detection is completed. Aiming at the problem that a large amount of dust often obstructs the visual field in the process of ore transportation, a multi-level feature fusion de-fogging network FFA-Net^16^, which combines channel attention and pixel attention, was introduced, and a composite and scalable ore feature extraction trunk was constructed with MBConv^17^ module based on depth-separable convolution. This is shown in the backbone section of Fig. 1. Aiming at the problems of the dense accumulation of blasting ore and drastic changes in particle size, a neck feature fusion network combining convolution and serial model-based Vmamba^18^ was introduced, and BiFPN^19^ cross-path bidirectional feature fusion architecture was adopted. This is shown in the neck section of Fig. 1. To solve the problem that the edge of the ore wet for dust suppression is not clear, and the conventional detection and segmentation methods can cause easy fusion with the surrounding ore, thus affecting the particle size calculation, the multidimensional feature fusion capability of Dynamic Head^20^ was introduced to optimize the target detection head and further optimize the feature fusion so that the feature tensor obtained from the ore image can be adapted to the ore detection and positioning task.

The overall algorithm pseudocode is shown in Algorithm 1:

**Algorithm 1:**
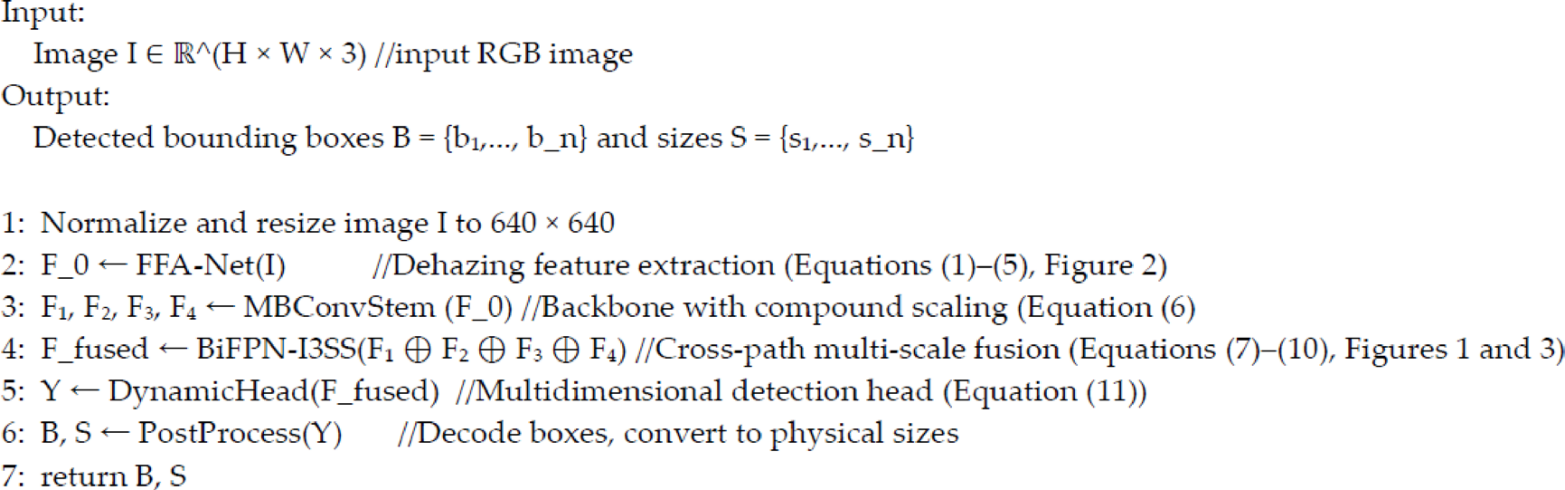
End-to-end ore size detection pipeline.

1. Efficient dust removal backbone

In this study, an efficient feature extraction backbone was constructed and combined with the FFA-Net image dust removal feature fusion network, as shown in the backbone part in Fig. 1; the model is optimized for scenes with large amounts of dust to improve the accuracy of rock detection. In the ore particle size detection, a large number of suspended particles, such as smoke, smoke, and mist, are produced by rock fragmentation, which easily causes image color distortion, blurring, and contrast reduction, which significantly affect the quality of the image. This degradation of image quality increases the difficulty of the target detection task and may even lead to a large number of gravel target detection failures. In order to solve this problem, image preprocessing such as enhancement is needed. Hayat^21^ applied attentiveness-guided super-resolution to the whole slide image under challenging visual noise, enhanced the fine-grained texture, and improved the quality of super-resolution image. Inspired by this, the FFA-Net dedusting network is introduced into the model structure to preprocess the input damaged image so as to restore a clearer image and assist the realization of subsequent detection tasks.

**Fig. 2:**
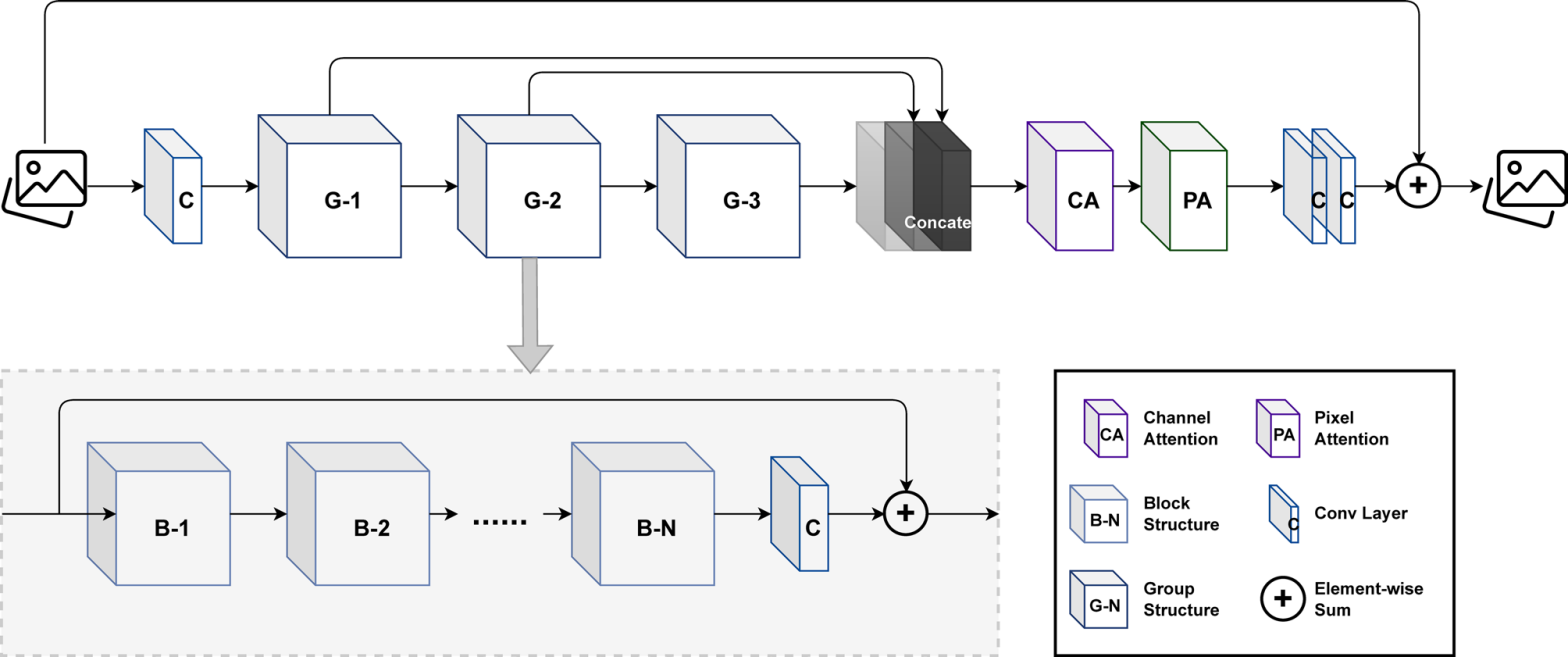
FFA-Net image dust removal feature fusion network structure.

The FFA-Net module in the backbone of Fig. 1 is shown in Fig. 2. The FFA-Net image dust removal feature fusion network is composed of a shallow feature extraction module, multiple groups of local residual structures, feature attention module (FA), and image reconstruction module. Channel attention (CA) and pixel attention (PA) mechanisms were fused to realize image sharpness and high-frequency detail enhancement. Figure 2 shows the detailed module composition of the network and its data flow direction. The input is a fuzzy image, which is first processed by a shallow feature extraction module and then passed into an N-group architecture containing multiple sets of jump connections. In these architectures, the Feature Attention (FA) module is used to fuse the output features of each group, and then, the fused features are passed to the reconstruction module and the global residual learning structure to generate clear, fog-free image output. Each set of architectures consists of a B-basic block and a local residual learning module in which the basic block combines a jump connection with a feature attention mechanism. Local residual learning within a basic block bypasses secondary information such as mist or low-frequency regions by multiple local residual connections, allowing the main network to focus on more critical feature information. The Feature Attention module consists of the CA Channel Attention and Pixel Attention mechanism, which provide greater flexibility in processing different types of information.

The embedded CA attention aims at the channel information and the target coordinate information related to the direction and weaves the coordinate dimension information into the channel attention mechanism to obtain information in a wider area. This attention mechanism adopts a segmentation strategy, which is concreted into a parallel process focusing on one-dimensional feature encoding to reduce the loss of location information caused by 2D global pooling. The CA module uses two pooling kernels of different sizes, (1, W) and (H,1), to encode the horizontal and vertical spatial coordinates of each channel, respectively, so that the module efficiently aggregates features in two spatial dimensions and ensures that the generated feature map retains key location information. The CA module internally uses average pooling for spatial encoding along the horizontal and vertical dimensions (Eq. (1)), followed by convolution and activation (Eq. (2)) and finally applying location-aware weights to the original feature maps (Eq. (3)).

1 $$\left\{ {\begin{array}{*{20}l} {z_{c}^{h} (h) = \frac{1}{W}\sum\limits_{{0 \le i \le W}} {x_{c} } (h,i)} \hfill \\ {z_{c}^{w} (w) = \frac{1}{H}\sum\limits_{{0 \le j \le H}} {x_{c} } (j,w)} \hfill \\ \end{array} } \right.$$

2 $$\left\{ {\begin{array}{*{20}l} {f = \delta \left( {F_{1} \left( {\left[ {z^{h} ,z^{w} } \right]} \right)} \right)} \hfill \\ {g^{h} = \sigma \left( {F_{h} \left( {f^{h} } \right)} \right)} \hfill \\ {g^{w} = \sigma \left( {F_{w} \left( {f^{w} } \right)} \right)} \hfill \\ \end{array} } \right.$$

3 $${y_c}(i,j)={x_c}(i,j) \times g_{c}^{h}(i) \times g_{c}^{w}(j)$$

where $z_{c}^{h}$ and $z_{c}^{w}$ (indexed by height and width) are the output values of the c channel. $\:f$ is the intermediate feature, and $\:{F}_{h}$ and $\:{F}_{w}$ are the corresponding convolution operations. $\:\delta\:$ is used to introduce nonlinear elements, $[ \cdot , \cdot ]$, to perform feature concatenation operations in the spatial dimension. The sigmoid activation function $\:\sigma\:$ performs a nonlinear transformation of the intermediate features to map them to the interval [0, 1], which generates the weights $\:{g}^{h}$ and $\:{g}^{w}$. Eventually, these weights are combined with the original feature map to produce the output of the CA module $\:{y}_{c}$.

Because of the uneven distribution of gravel dust on image pixels, Pixel Attention (PA) focuses more on extracting specific information features, such as pixels in dusty areas and detailed parts of high-frequency images. PA is processed by passing the input of channel attention (CA) to two convolutional layers, which use ReLU and Sigmoid as activation functions, respectively. The inner Conv + ReLU ($\:\delta\:$) is used to extract and amplify salient features (such as dust gathering areas or high-frequency edges) from the channel attention output $\:{y}_{c}$, while the outer Conv + Sigmoid ($\:\sigma\:$) maps these features into a spatial attention weight map of [0,1], which is used to do pixel-by-pixel weighting of the original channel-layer features. The function of the two steps can be understood as: first, the important locations are extracted/enhanced, and the normalized gated weights are regenerated. Finally, the data shape is transformed from C × H × W to 1 × H × W.

4 $$PA=\sigma (Conv(\delta (Conv({y_c}))))$$

Finally, element-wise multiplication of ${y_c}$ and PA is performed, where Fa is the output of the FA module.

5 $${F_a}={y_c} \otimes PA$$

The FFA module is jointly optimized with the rest of the network during training. The FFA-Net dust removal network is used to preliminarily process the captured rock image, which makes the high-frequency components in the image more obvious and highlights the details in the original image. After dust removal, the rock object in the image is clearer, the contour boundary is more obvious, and the object contrast is stronger, which can improve the recognition accuracy of the object detection algorithm.

The backbone network is very important to the detection task. Peng et al.^22^ introduced the BiFormer attention mechanism to redesign the Efficientv2 network, and obtained a novel backbone network for efficiently extracting the features of students ‘classroom behavior. Wang et al.^23^ replaced the feature extraction module in the EfficientNet-B3 core with an improved feature extraction (MBConv + +) module to better capture the dependencies between channels. Inspired by this, we input the clearer feature map obtained from the original image after dusting through the FFA-Net network into the efficient multi-level feature extraction backbone network constructed by MBConv basic block that can be composite scaled. From low level to high level, the sensitivity field of the ore image is expanded step by step, and the ore features of various sizes are comprehensively captured. The basic block of MBConv firstly performs a 1 × 1 convolution operation to expand the number of channels of the input ore feature map and then uses an efficient depthwise separable convolution operation to extract ore edge, texture, and other features. Finally, it performs a 1 × 1 convolution operation to reduce the number of channels of the ore feature map for subsequent modules to calculate. In this process, the channel expansion rate and compression rate of each MBConv basic block can be adjusted so that the whole network can be composite-scaled, and then, the network structure with the highest efficiency and the best performance of ore image feature extraction is found through grid search to construct the efficient dust feature extraction backbone network. The overall network is expressed as follows:

6 $$Backbone=F_{n}^{{{L_n}}}( \cdots F_{2}^{{{L_2}}}(F_{1}^{{{L_1}}}({X_{{H_1} \times {W_1} \times C_{1}^{{{E_1}}}}})))=\prod\limits_{{i=1}}^{n} {F_{i}^{{{L_i}}}({X_{{H_i} \times {W_i} \times C_{i}^{{{E_i}}}}})}$$

where Hi × Wi is the resolution of the input ore feature map, Ci is the number of channels of the input ore feature map, X is the input information of the operation Fi, Ei is the dimension magnification of the 1 × 1 convolution, Fi is the operation corresponding to the network layer i, and Li is the number of network layers of the operation Fi.

The MBConv basic block parameters of each layer after composite scaling of the backbone network are shown in Table 1.

**Table 1:** Backbone network structure.

Network layer i	The resolution Hi × Wi	Operate Fi	Dimension magnification Ei	Number of output channels Ci	The number of network layers Li
1	640 × 640	FFA-Net	–	3	1
2	640 × 640	MBConv3 × 3	1	16	1
3	320 × 320	MBConv3 × 3	6	24	3
4	160 × 160	MBConv5 × 5	6	40	3
5	80 × 80	MBConv3 × 3	4	80	4
6	40 × 40	MBConv5 × 5	4	112	4
7	40 × 40	MBConv5 × 5	4	192	5
8	20 × 20	MBConv3 × 3	4	320	2

(2) The I3SS feature fusion module is introduced in the neck

**Fig. 3:**
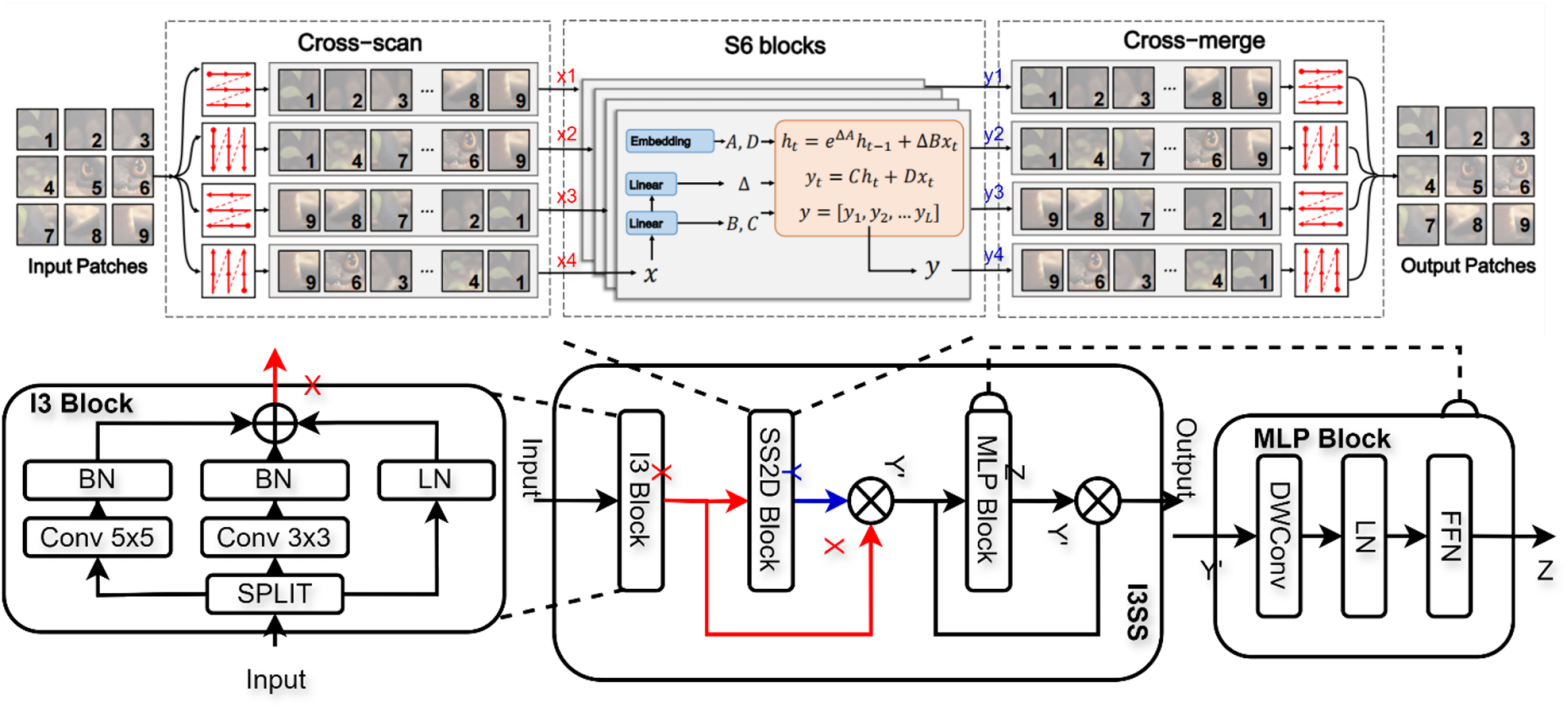
Network architecture of I3SS block.

The modules in the neck network are mainly designed to extract and fuse multi-scale mineral features from backbone feature maps of different resolutions. The feature extraction and fusion performance of the module will affect the subsequent ore positioning accuracy and the accuracy of distinguishing between ores and background. The C2f module used in the original YOLOv8 is suitable for conventional target feature extraction, but its performance is insufficient in blasting ore detection tasks under various environmental disturbances. Blasting rock detection requires capturing both local texture details (such as the edges and contours of small rocks) and global context relationships (such as the separation between overlapping or stacked rocks), and to detect fine-grained rocks, higher-resolution feature maps need to be processed, while mining sites often have limited computing resources. Vmamba can capture long-range spatial dependencies with linear computational complexity, which is particularly suitable for high-resolution feature maps and dense rock stacking scenarios. Compared with the transformer-based attention mechanism, Vmamba provides global context modeling while significantly reducing memory and running time costs. Compared with recurrent or pure convolutional sequence models, it can provide more stable and efficient modeling of long-range dependencies. Therefore, this paper introduced the I3SS module combining the convolution model and Vmamba model based on the sequence model instead of the original C2f module to integrate multi-scale ore features, as shown in the neck part of Fig. 1. In our I3SS design, Vmamba is used to supplement the convolutional local feature extraction, which helps to separate closely adjacent minerals, and improves the recognition ability of small objects in the presence of dust and occlusion. At the same time, it provides a good foundation for the accurate tracking and statistics of mineral targets in the video. Figure 3 shows the network structure of the I3SS module, the I3SS module is composed of three parts: I3, SS2D and MLP.

After the blasting ore feature tensor extracted from the backbone network is input into the I3SS module, the I3 module is first used for convolution operation. The I3 module uses convolution kernels of two sizes to process the ore feature tensor and concatenate it with the original features respectively. The two kernel sizes provide complementary local receptive fields: 3 × 3 captures fine high-frequency details (good for small objects), while 5 × 5 captures larger context. Learn combinations during training: The convolutional weights are optimized so that for different image content, one or other convolutional paths will contribute stronger activations in the fused tensor. In practice, this leads to adaptive effective receptive fields even when the kernel size itself is fixed. In order to accelerate model convergence and enhance model stability, batch normalization is employed. This not only reduces overfitting but also provides a certain degree of regularization effect, and the intermediate state I3 thus obtained is defined as:

7 $${I_3}=Bn\left( {conv5\left( I \right)+conv3\left( I \right)+Ln\left( I \right)} \right)$$

The intermediate state I3 combines channel information through a 1 × 1 convolution and better maintains the distribution of information through an activation function, enabling the model to learn more complex feature representations. These feature representations can extract rich multi-scale context information from the input feature maps. In the I3SS Block, the activation function uses the nonlinear GELU to change the number of feature channels without altering the spatial dimension, thereby enhancing the feature representation. Finally, the original input is fused with the processed features through residual concatenation. This enables the model to understand and integrate features of different dimensions in the image, thereby improving its robustness to scale variations. The ore characteristic tensor after convolution and concatenation of the I3 module is input into the SS2D module for further calculation. The SS2D module is divided into three parts: Scan Expand, S6 Block and Scan Merge. The core of the SS2D module is the state space model Mamba (S6) for processing sequence data^24^. Since the S6 module processes sequential data, and the ore images and the extracted feature tensor data have three dimensions, SS2D will perform Scan Expand on the ore feature tensor along four different directions (from left to right, from right to left, from top to bottom, and from bottom to top) in the H×W dimensions, obtaining four different pixel sequences x1 to x4, which are then input into the S6 module. The S6 state transformation formula is as follows:

8 $${h_i}=A{h_{i - 1}}+B{x_i}$$

9 $${y_i}=C{h_i}+D{x_i}$$

x is the pixel sequence generated by the Scan Expand module. h is the current status information. A is the matrix of state transition, representing the dynamic characteristics of state change. B is the input control matrix, representing how input x affects the state. y is the output feature sequence. C is the state control matrix, representing how state h controls the output. D is the direct transmission matrix, representing the direct influence of input x on output y. During the model training process, the S6 state matrix (A, B, C, D) and the internal state dynamics were learned. They model long-range dependencies and context of spatially distant pixels. This allows the network to adaptively adjust the local response based on larger-scale structure (e.g., whether a pixel belongs to a small isolated mineral blocks or is part of a large cluster). Finally, Scan Merge will re-arrange the four output state sequence y1 to y4 in the order of scanning to form a new feature image, and combine the four obtained feature images to form an output feature image with the same dimension as the input image. Since each feature pixel of the feature image is integrated from all other pixels in different directions, and each pixel point is selected and filtered according to the input information by the Selective State Space Models (S6), both matrix B(x) and matrix C(x) change dynamically according to the pixel sequence x. Therefore, each feature pixel not only comprehensively covers all areas of the input image, can effectively capture distant pixel dependencies, but also provides a rich multi-dimensional information library for the extraction of feature pixels through the system’s direction transformation, thereby improving the efficiency and comprehensiveness of the multi-dimensional capture of image features. The combination of I3 and SS2D module can effectively fuse the local features such as texture edge of a single ore, and separate the features of different ores, which is conducive to the detection of ore targets. Finally, the output ore feature tensor is nonlinear transformed and mixed by the MLP module. This MLP effectively re-weights and mixes the multi-scale contributions on the basis of each channel (and each position after the spatial operator) to enhance the expression ability of the model. The calculation formula of the MLP module is as follows:

10 $$MLP=FFN\left( {LN\left( {DWConv\left( Y \right)} \right)} \right)$$

The I3SS parameters and the rest of the detection model are trained end-to-end using the standard detection targets used in our experiments, which are the same as those in YOLOv8 basic training. Specifically, the following learnable components in I3SS are optimized:

1. Convolution kernels in conv3 and conv5 (I3) : weight + bias.
2. LayerNorm/BatchNorm affine parameters used in the fusion.
3. S6/Vmamba state space matrix and input/output projection matrices (A, B, C, D) and any associated linear layers in SS2D.
4. The depth conv + MLP(FFN) weights that produce the final transformed feature MLP(Y).
5. Downstream detection head weights (dynamic heads) jointly optimized with I3SS.

All of the above are jointly trained by minimizing the detection loss (classification loss, objectness loss, and bounding box regression loss—the sum of the CIoU/IoU variants used in our implementation).

(3) Neck cross-path bidirectional feature fusion architecture

The YOLOv8 original neck network fuses the three feature maps extracted from the three low-resolution feature layers 80 × 80, 40 × 40, and 20 × 20 in the upper layer of the backbone and then uses up-sampling and convolution down-sampling, respectively, to form a bidirectional path to fully fuse different feature information in the high-low resolution layer. This structure is suitable for general target detection, but the blasting ore target has a drastic scale change, and a lot of small-sized ore characteristic information is lost in the feature map of the medium and low resolution of the high-rise. Moreover, due to the excessive length of the bidirectional path, the blasting ore characteristic information needs to cross the transverse connection of at least two paths from the main body to the detection head, and a lot of fine ore characteristic information will be lost in this process. Thus, the ore detection rate is decreased. Therefore, the cross-path bidirectional feature fusion architecture of the neck was introduced, which introduces the 160 × 160 resolution feature map containing rich detail information of the fine ore in the neck. At the same time, the cross-path connection directly from the trunk to the outermost path is increased, and the loss of fine ore, ore edge, and texture details is reduced. Combined with the I3SS module, the model can effectively improve the ability to detect and locate small-sized ores.

(4) Target detection head optimization

The Dynamic Head applies the attention mechanism to multiple dimensions of the blasting ore feature tensor processed by the feature fusion network based on the neck I3SS module and using the cross-path bidirectional feature fusion architecture, as shown in the Dynamic Head section in Fig. 1. The edge and texture feature information of each particle size ore in the feature tensor becomes clearer, the model pays more attention to the location of the ore, and the ore feature tensor of the final input detection head is more suitable for the tasks of ore target location, ore target, and background and other ore discrimination. The detector has better detection ability for all sizes of ore in the scene where ore is densely stacked, and the edge is not clear after wetting. The Dynamic Head applies the attention calculation formula to the blasting ore feature tensor F, as shown in Eq. (4). In contrast to decoupled heads (such as DETR style Query or RT-DETR Dynamic sparse heads), Dynamic Heads provide a lightweight, task-aware attention mechanism that allows the model to dynamically adjust to scale, spatial, and task-level cues without the need for transformer-based global context modeling. This can be computationally expensive and sensitive to data size. Since our images come from mining environments, with dense, small objects and scale changes and the need to process higher-resolution feature maps, Dynamic Head improves performance while reducing FPS degradation during model inference, making the model usable in preset scenarios.

11 $$W\left( F \right)={\pi _C}\left( {{\pi _S}\left( {{\pi _L}\left( F \right) \cdot F} \right) \cdot F} \right) \cdot F$$

### Experimental environment

1. Ore granularity detection dataset

**Fig. 4:**
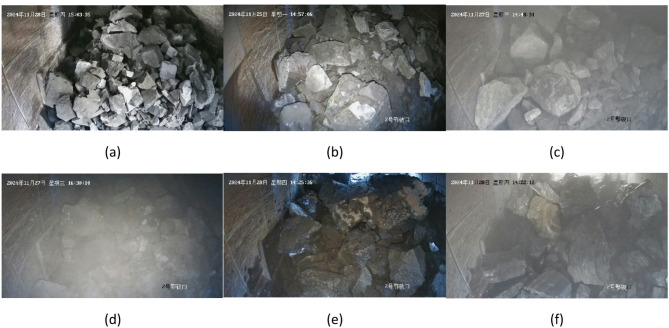
Examples of typical scenarios. The top-left and bottom-right corners of each image are the time and location watermarks, which do not affect the model training and detection. (**a**) Natural Light, (**b**) Lamplight, (**c**) Light Dust, (**d**) Heavy Dust, (**e**) Wet Ore, (**f**) Wet Ore and Raises Dust.

The ore pictures were collected from a high-definition camera with a resolution of 3840 × 2160, and the scene comprised the ore on the feed port and conveyor belt of the jaw crusher. The footage of the jaw break feed video recorded in the real production environment of the mine was disassembled, and the ore data of the jaw break blasting were obtained. The semi-automatic labeling tool based on open-set target detection was used for preliminary labeling of the detection frame. The preliminary annotated data were then manually fine-tuned and supplemented. We used calipers to manually record real physical measurements corresponding to samples of partially annotated images to convert pixel sizes to real sizes. The ore particle size is estimated according to $\:\boldsymbol{d}=\sqrt{\boldsymbol{w}\mathbf{*}\boldsymbol{h}}$ (w, h are the object bounding box width and height, respectively). For the pictures with different degrees of dust, we enlarged the pictures and manually labeled all the minerals as far as the human eye could distinguish. A total of 356 pictures were collected and marked for training and verification. Among them, the lighting environment can be divided into natural light during the day and night lighting, and the ore state can be divided into dry and wet. There may be different concentrations of smoke in the scene, which makes the vision affected or even completely obscured. The above environment and ore conditions may be combined, and various scenarios are evenly distributed in the training set and verification set. Typical scenarios are as shown in Fig. 4.

**Fig. 5:**
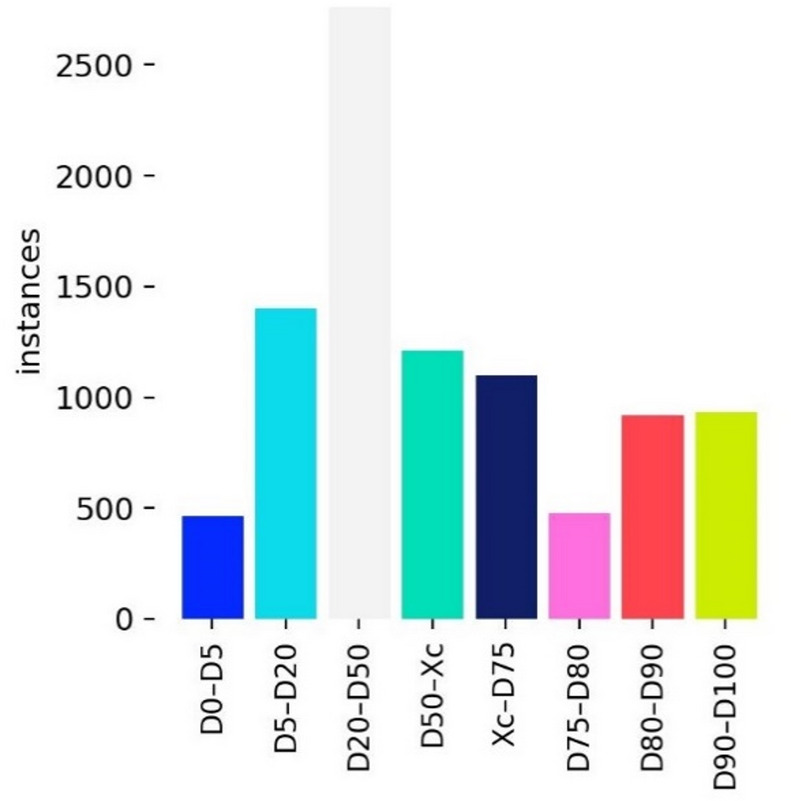
Number of instances in each category.

The dataset was split with a ratio of train/val = 9:1, and the scenes in the training and validation sets are evenly distributed. In order to better evaluate the detection performance of the algorithm for ores of various particle size sizes, we classified the target boxes in the labeled jaw break blasting ore detection dataset according to particle size and used them for subsequent experiments. We took the ore target frame within a certain particle size range as a class and divided the ore target frame into eight classes according to the particle size number fraction: D0–D5, D5–D20, D20–D50, D50–Xc, Xc–D75, D75–D80, D80–D90, and D90–D100. The sample number of each category is shown in Fig. 5. To measure the detection performance of the detector for ores in various particle size ranges instead of describing the ore mass distribution, we used quantity scores instead of quality scores. Among them, the D5 particle size value represents the 5% number of ore target boxes with a particle size smaller than this value, D5–D20 represents the ore target boxes with a particle size between D5 and D20, Xc represents D63.2, and the other seven categories are similar. The number of targets in each category is shown in the following figure. Since ores in each category are only different in size, there is no significant difference in characteristics. The classification of ore target boxes according to particle size is only to facilitate the demonstration of the model’s detection performance improvement for ores with different particle sizes, and classification does not affect particle size detection.

(2) Experimental environment and parameter setting

The experimental environment and parameter settings are shown in Table 2.

**Table 2:** Experimental environment and parameter setting.

Environment/parameters	Value	Environment/parameters	Value
OS	Ubuntu 20.04.6	Initial learning rate	0.01
CUDA	11.8	Final learning rate	0.01
Python	3.10.15	Momentum	0.937
Pytorch	2.1.1	Optimizer weight decay	0.0005
Optimizer	auto	Warmup-epochs	3.0
Batch	8	Warmup-momentum	0.8
Epochs	150	Warmup-bias-lr	0.1
Image size	640 × 640	Mosaic augmentation	10

(3) Evaluation metrics

The evaluation metrics selected in the experiment in this paper include accuracy rate P, recall rate R, average accuracy mean mAP, and detection frames per second FPS to measure the model’s detection performance and effect on ores of different particle sizes in the dataset and reflect the pros and cons of each model. The calculation formula is as follows.

12 $$P=\frac{{TP}}{{TP+FP}}$$

13 $$R=\frac{{TP}}{{TP+FN}}$$

14 $$\begin{array}{*{20}{c}} {mAP=\frac{1}{c}\mathop \sum \limits_{{i=1}}^{c} A{P_i}} \end{array}$$

15 $$mAE=\frac{1}{N}\mathop \sum \limits_{{i=1}}^{N} \left| {{{\hat {y}}_i} - {y_i}} \right|$$

where TP represents the amount of ore that is present and predicted to be ore, and FP represents the amount of ore that is not present but predicted to be ore. FN indicates the amount of ore that is absent but predicted to be present at that location. APc represents the average precision of each class, and c represents the number of all ore particle size categories. mAP50 measures the average AP of the model when the IoU threshold is 0.5. mAP50 − 95 measures the average AP of the model over a range of IoU thresholds from 0.5 to 0.95. $\:{\widehat{y}}_{i}$ is the ore particle size predicted by the model, $\:{y}_{i}$ is the real labeled particle size value, N is the number of samples, and mAE (mean absolute error) index measures the numerical error between the predicted ore particle size and the real size.

## Results

### Comparative experiments

To evaluate the performance of the improved model, this paper compares the proposed blasting ore particle size detection model based on efficient dust removal network and multi-dimensional feature fusion with a variety of mainstream target detection models on the produced blasting ore detection dataset on NVIDIA GeForce GTX 3060 equipment. The input image resolution is 384 × 640 for 150 batches of training, and the experimental results are shown in Tables 3, 4, 5 and 6. In the table, $\overline {P}$, $\overline {R}$, and mAP50 all represent the average values of the eight classes.

**Table 3:** Comparison of mainstream model checking results.

Method	Backbone	$\overline {P}$ (%)	$\overline {R}$ (%)	mAP50 (%)	FPS
ATSS^25^	ResNet-50	42.0	65.4	53.8	43
AutoAssign^26^	ResNet-50	52.4	61.3	52.4	47
Faster-RCNN^27^	ResNet-101	46.6	58.8	52.2	36
Localization Distillation^28^	ResNet-18	44.7	59.4	52.0	53
FoveaBox^29^	ResNet-50	41.0	58.9	49.1	50
TOOD^30^	ResNet-50	50.1	**66.3**	54.6	47
FCOS^31^	ResNet-101	45.1	59.9	52.1	41
Generalized Focal Loss^32^	ResNet-50	49.8	65.9	55.3	39
YOLOv8n	CSPDarknet-53	45.7	51.2	48.7	**268**
Improved Algorithm	FFA-efficientNetV1	**52.7**	57.8	**56.3**	26

Bold letters indicate optimal results.

The improved algorithm shows good detection performance on the performance metrics $\overline {P}$ and mAP50, surpassing all other algorithms, improving by 0.3% and 1%, respectively, compared with the suboptimal algorithm. Compared with the same single-stage strategy, the ATSS with accuracy $\overline {P}$ and mAP50 metrics improved by 10.7% and 2.5%, respectively, compared with the two-stage method with deeper network, Faster-RCNN, which improved by 10.7% and 2.5%, respectively. The recall rate $\overline {R}$ of YOLOv8n is lower than that of other methods. The reason may be that the fixed number of anchor boxes is used to predict the target. This design and matching strategy may not be flexible in some cases, especially when the target of multiple different sizes and shapes is not well matched with the predefined anchor boxes. This leads to many missed detection problems. By improving YOLOv8n, the recall rate $\overline {R}$ of our algorithm reaches the general level of mainstream models. The inference speed of the algorithm is measured in FPS (number of image frames processed per second). Localization Distillation uses ResNet-18 as the base network and has fewer convolutional layers, thus reasoning faster than other networks using ResNet-50. Although the performance metrics accuracy of $\overline {P}$, recall rate of $\overline {R}$, and mAP50 of YOLOv8n are lower than most algorithms, the reasoning speed is far higher than other algorithms, and the FPS is four to seven times that of other algorithms. The improved algorithm improves the detection performance at the cost of a partial decrease in FPS, which is in line with the characteristics of blasting ore detection that emphasizes detection accuracy and ignores detection speed.

The detection performance indicators of YOLOv8n for each particle size class are shown in Table 4.

The detection performance metrics of each particle size class of the improved algorithm are shown in Table 5.

The values of various performance indicators of the improved algorithm relative to YOLOv8n are shown in Table 6.

**Table 4:** YOLOv8n detection performance metrics.

Class	All*	D0–D5	D5–D20	D20–D50	D50–Xc	Xc–D75	D75–D80	D80–D90	D90–D100
$\overline {P}$ (%)	45.7	24.4	43.5	50.9	40.1	40	36.1	60.2	70.6
$\overline {R}$ (%)	51.2	7.84	45.6	58.2	48.6	51.8	32.5	77.6	87.8
mAP50 (%)	48.7	13.4	41	56.4	44.2	46.3	27.3	74.2	86.9
mAP50–95 (%)	35.3	7.76	26	37.3	33.2	33.7	20.3	57.7	66.5
mAE (mm)	67.68	64.47	57.71	58.53	53.4	81.63	96.32	58.89	70.52

*All denotes the average of the metrics over all classes.

**Table 5:** Improved algorithm detection performance metrics.

Class	All*	D0–D5	D5–D20	D20–D50	D50–Xc	Xc–D75	D75–D80	D80–D90	D90–D100
$\overline {P}$ (%)	52.7	43.2	46	56.6	50.6	49.7	34	64.9	76.3
$\overline {R}$ (%)	57.8	21.6	54.4	66.6	64.1	60.7	37.5	72	85.7
mAP50 (%)	56.3	26.9	52.5	66.5	62.2	52.3	27.3	74.1	88.3
mAP50–95 (%)	43.3	14.4	38	50.2	52.6	41.7	21.5	60.1	67.6
mAE (mm)	57.42	57.82	49.63	42.55	34.25	63.6	98.76	49.35	63.42

*All denotes the average of the metrics over all classes.

**Table 6:** Detection performance gains after improvement.

Class	All*	D0–D5	D5–D20	D20–D50	D50–Xc	Xc–D75	D75–D80	D80–D90	D90–D100
$\overline {P}$ (%)	7	18.8	2.5	5.7	10.5	9.7	−2.1	4.7	5.7
$\overline {R}$ (%)	6.6	13.76	8.8	8.4	15.5	8.9	5	−5.6	−2.1
mAP50 (%)	7.6	13.5	11.5	10.1	18	6	0	−0.1	1.4
mAP50–95 (%)	8	6.64	12	12.9	19.4	8	1.2	2.4	1.1
mAE (mm)	−10.26	−6.65	−8.08	−15.98	−19.15	−18.03	2.44	−9.54	−7.1

*All denotes the average of the metrics over all classes.

It can be seen from the above table that the detection performance metrics of fine ore below D5 show the most obvious improvement, with the accuracy rate P increased by 18.8% and the recall rate R increased by 13.8%. The detection performance metrics of ore with particle size below D75 are significantly improved, and the detection performance metrics of ore with particle size below D75 are slightly improved or slightly decreased.

In order to verify the cost effectiveness of our model, the proposed model was compared with other recent lightweight detectors, and the results are shown in Table 7. Our model outperformed other models in metrics such as P, mAP50, mAP50–95, and mAE and was only slightly lower than the best YOLO11s in recall P. The cost of GFLOPS and RAM of our model is slightly higher than that of YOLOv10s and YOLO11s, and most performance indicators significantly improved. The FPS of our model is lower than other lightweight detectors, but as a model used to detect the ore size before the jaw break, FPS is not the primary consideration due to the slow transmission speed of the conveyor belt, and the FPS of our model can meet the requirements. And when the model was actually applied to the dynamic detection and tracking of ore on the conveyor belt, we found that although the FPS of YOLOv8, YOLOv10, YOLO11, and other models is very high, the detection frames generated by them in consecutive video frames are less stable and often produce a certain random offset, which leads to the loss of ore during tracking and eventually double counting. It seriously affects the accuracy of particle size detection, which is a disadvantage that cannot be ignored for ore particle size detection. However, the target frame generated by our model has good stability between video frames, and the problem of missing ore targets is greatly improved. Our model outperforms transformer-based models such as RT-DETR and YOLOv8n based on Swin Transformer backbone in all performance metrics, which may be due to the large computational overhead and slow convergence speed of transformer.

**Table 7:** Comparison with recent lightweight detector performance metrics.

Model	$\overline {P}$ (%)	$\overline {R}$ (%)	mAP50 (%)	mAP50–95 (%)	mAE (mm)	GFLOPs	RAM (MB)	FPS
YOLOv8n	45.7	51.2	48.7	35.3	67.68	8.1	6.2	**268**
YOLOv10n^33^	40.3	49.5	41.4	29.6	116.18	8.4	5.7	173
YOLOv10s	43.3	54.1	47.7	36.5	88.75	24.8	16.5	166
YOLO11n	40.4	54.9	47.2	33.9	79.21	**6.4**	**5.5**	174
YOLO11s	47.4	**58.4**	52.7	39.8	58.44	21.6	19.2	172
YOLO12n^34^	40.8	55.1	46.1	32.5	75.18	6.5	5.5	116
YOLO12s	51.2	56.9	54.7	41.1	60.54	21.5	18.9	123
RT-DETR ResNet50	3.11	11.4	3.09	0.757	257.06	130.5	86.1	53
RT-DETR-l	6.34	2.28	3.25	1.61	257.06	105.2	59.1	63
YOLOv8n-SwinTransformer	37.7	56.3	44.6	31.3	86.23	79.1	60.5	100
Improved Algorithm	**52.7**	57.8	**56.3**	**43.3**	**57.42**	29.4	22	26

Bold letters indicate optimal results.

**Fig. 6:**
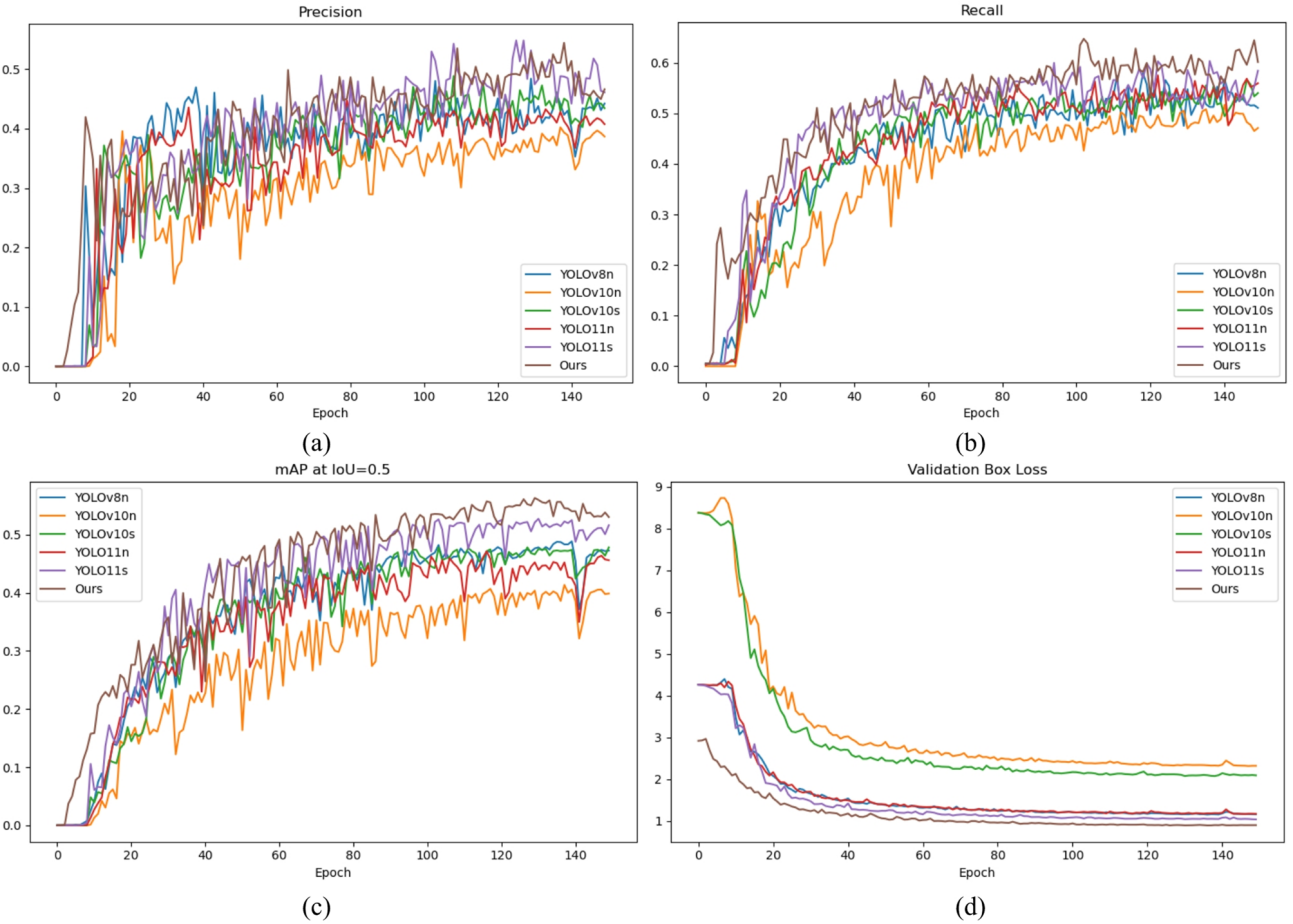
Metrics/loss–epoch curve in train/test. (**a**) precision–epoch, (**b**) recall–epoch, (**c**) mAP50–epoch, (**d**) loss–epoch.

In order to better test the performance of the proposed algorithm, we compared the changes of metrics and loss of the proposed algorithm with the recent lightweight detectors during the training process. The precision–epoch, recall–epoch, mAP50–epoch, and loss–epoch curves are plotted as shown in (a–d) in Fig. 6, respectively, and the curves further show that our algorithm outperforms the recent lightweight detectors.

Finally, experiments were carried out on the total one-class dataset to verify the overall detection performance metrics of the algorithm for blasting ore. The input image resolution is 1440 × 2560, and 150 batches of training were carried out. The experimental results are shown in Table 8.

**Table 8:** Comparison of detection performance metrics on single-class dataset.

Model	*P* (%)	*R* (%)	mAP50 (%)	mAP50–95 (%)	mAE (mm)
YOLOv8n	**81**	77.2	86.6	70.7	38.95
Improved algorithm	75.6	**84**	**88.1**	**72.8**	**30.56**

Bold letters indicate optimal results.

On the total one-class dataset, the recall rate of the improved algorithm was 84%, and the mAP50 was 88.1%. Compared with the YOLOv8n algorithm, the recall rate R, mAP50, and mAP50–95 increased by 6.8%, 1.5%, and 2.1%, respectively, and the mAE decreased by 8.39 mm. This shows that the recognition rate and positioning accuracy of the improved algorithm are significantly improved, and the overall error of particle size recognition is significantly reduced.

### Ablation experiment

To verify the effectiveness of the improved three parts of the YOLOv8n network proposed in this paper, aiming at the characteristics of blasting ore particle size detection, we conducted ablation experiments based on the YOLOv8n model on the aforementioned ore particle size detection dataset, controlled whether three improvement points were added or not, and evaluated the influence of different improvement points on the performance of the blasting ore particle size detection algorithm and their mutual influence. The results of the ablation experiments are shown in Table 9.

**Table 9:** Data table of ablation experimental results.

Optimize backbone	Optimize neck	Optimize detection head	$\overline {P}$ (%)	$\overline {R}$ (%)	mAP50 (%)	mAP95 (%)	mAE (mm)
			45.7	51.2	48.7	35.3	67.68
√			46	54.6	50.6	37.4	63.12
	√		43.2	58.3	50.4	37.2	67.27
		√	43.7	56.9	49.6	35.7	77.76
√	√		47.9	54.9	52.3	39.5	67.24
√		√	48.2	55.2	52.3	38.6	62.41
	√	√	46.9	61.8	54.7	41.8	60.05
√	√	√	52.7	57.8	56.3	43.3	57.42

Bold letters indicate optimal results.

As can be seen from the above table, the addition of trunk, neck, and detection head alone improved the mAP50 by 0.9–1.9% and the recall rate of $\overline {R}$ by 3.4–7.1%, and the accuracy rate of $\overline {P}$ was slightly increased or slightly decreased. Each surface optimization can effectively improve the blasting ore detection performance. With the combined application of two of them, mAP50 increased by 3.6–6%, the recall rate of $\overline {R}$ increased by 3.7–10.6%, and the accuracy rate of $\overline {P}$ increased by 1.2–2.5%, indicating that the combined application of each improvement point can further improve the blasting ore detection performance. The final ore detection performance metrics $\overline {P}$, $\overline {R}$, and mAP50 reached 52.7%, 57.8%, and 56.3%, respectively, which were 7%, 6.6%, and 7.6% higher than the benchmark.

To isolate the contribution of each component in the FFA-Net, MBConv backbone, and I3SS module and verify the effectiveness of the sequence model, we conducted ablation experiments inside the I3SS and add RT-DETR-based pure transformer neck as a comparison, and the results are shown in Tables 10 and 11 To verify the effect of I3SS on the detection performance of different particle sizes, we show the comparison of the performance index mAP50 of each category before and after the introduction of I3SS in the neck and the improvement of the index after its introduction, and the results are shown in Table 12.

**Table 10:** Ablation experiments inside the backbone network.

Model	*P* (%)	*R* (%)	mAP50 (%)	mAP50–95 (%)	mAE (mm)	GFLOPs	RAM(MB)	FPS
YOLOv8n	45.7	51.2	48.7	35.3	67.68	8.1	**6.2**	**268**
FFA only	44.6	56.1	49.5	35.5	77.85	13.2	6.3	164
MBConv Stem	43.7	**59.6**	49.4	35.6	70.91	**5.7**	20	103
FFA-MBConv Stem	**46**	54.6	**50.6**	**37.4**	**63.12**	10.8	20	80

Bold letters indicate optimal results.

**Table 11:** I3SS internal ablation experiments.

Model	*P* (%)	*R* (%)	mAP50 (%)	mAP50–95 (%)	mAE (mm)	GFLOPs	RAM(MB)	FPS
YOLOv8n	45.7	51.2	48.7	35.3	67.68	**8.1**	**6.2**	**268**
I3 only	41.8	58	49.3	36.2	73.25	14.7	11.6	211
SS2D only	**47.7**	56.9	50.1	35.9	74	12.6	8.8	135
RT-DETR	30.9	39.5	23	14.7	257.06	16.8	19.2	97
I3SS	43.2	**58.3**	**50.4**	**37.2**	**67.27**	16.7	11.6	85

Bold letters indicate optimal results.

**Table 12:** After the improvement, the detection performance index is increased.

Item	All	D0-D5	D5-D20	D20-D50	D50-Xc	Xc-D75	D75-D80	D80-D90	D90-D100
YOLOv8n	48.7	13.4	41	56.4	44.2	46.3	27.3	74.2	86.9
I3SS	50.4	23.2	41.3	59.1	50.9	47.2	25	68.6	88.3
Gains	1.7	9.8	0.3	2.7	6.7	0.9	-2.3	-5.6	1.4

All represents the average of the metrics over all classes.

The ablation experiments within the backbone network show that the FFA-Net and MBConv modules can improve some performance indicators, while other indicators slightly improve or decrease. The combination of the two modules has a better synergistic effect, and the final mAP50 is increased by 1.9% and mAE is decreased by 4.56 mm. At the same time, the backbone network optimization brings additional computational costs, but these costs are acceptable for our jaw break blasting ore granularity detection scenario. The results of I3SS internal ablation experiments show that both I3-only and SS2D-only neck feature fusion modules can bring detection performance improvement, while the pure Transformer neck model based on RT-DETR showed poor performance, which may be because the Transformer model is good at processing deep semantic features. However, the detection of a large number of small targets in the ore blasting image mainly relies on low-level detail features, Transformer has large computational overhead and slow convergence speed, and 150 epochs is not enough to converge. The experimental group of I3SS achieved the best detection results, confirming that combining local feature convolution (I3) with global sequence modeling (Vmamba SS2D) can produce a synergistic effect. Among all particle size categories, the performance index mAP50 has the largest improvement for small and medium size ores, and the ore with D0-D5 particle size has the largest improvement of 9.8%. Although I3SS introduces 8.6GFLOPs extra floating-point operations and 5.4 MB extra parameters, which reduces the FPS to one-third of the original FPS. However, ore crushing is a slow process, conveyor belts usually transport ore slowly, and jaw breakage ore size detection usually requires strict accuracy and relaxed speed requirements, so we believe that I3SS is cost-effective in our scenario.

### Visualization of experimental results

**Fig. 7:**
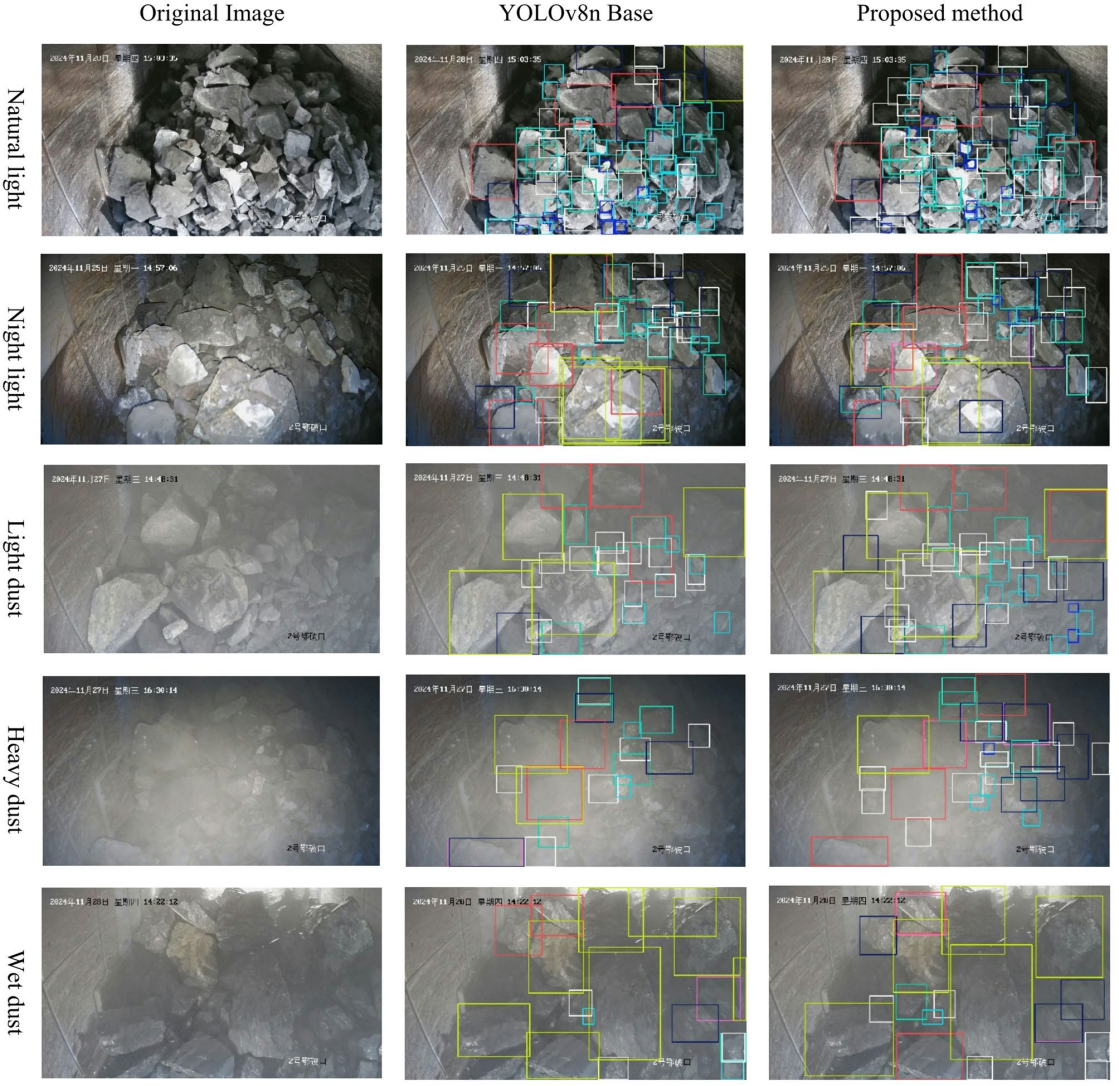
Comparison of detection effect. The top-left and bottom-right corners of each image are the time and location watermarks, which do not affect the model training and detection.

Under different lighting environments, ore states, and dust concentration environments, the YOLOv8n benchmark algorithm and improved algorithm were used to detect the image of jaw fracture ore, and the results were visualized as shown in Fig. 7. In an environment without dampness and smoke, the two models had the best detection effect, and the improved algorithm missed less ore and had a higher recall rate. Under the night light, the benchmark model showed obvious false detection of the ore, while the improved model did not show repeated detection boxes. In light and heavy dust, the improved model detected more ore than the benchmark model, and the detection rate of fine ore was higher. On the wet ore image, the improved model also detected more small-sized ore without the false check box in the upper right corner of the image. This shows that the improved algorithm has a higher accuracy for blasting ore detection, a higher recall rate for small-sized ore detection, and better robustness in detecting light and heavy dust, wet ore, and other disturbances.

**Fig. 8:**
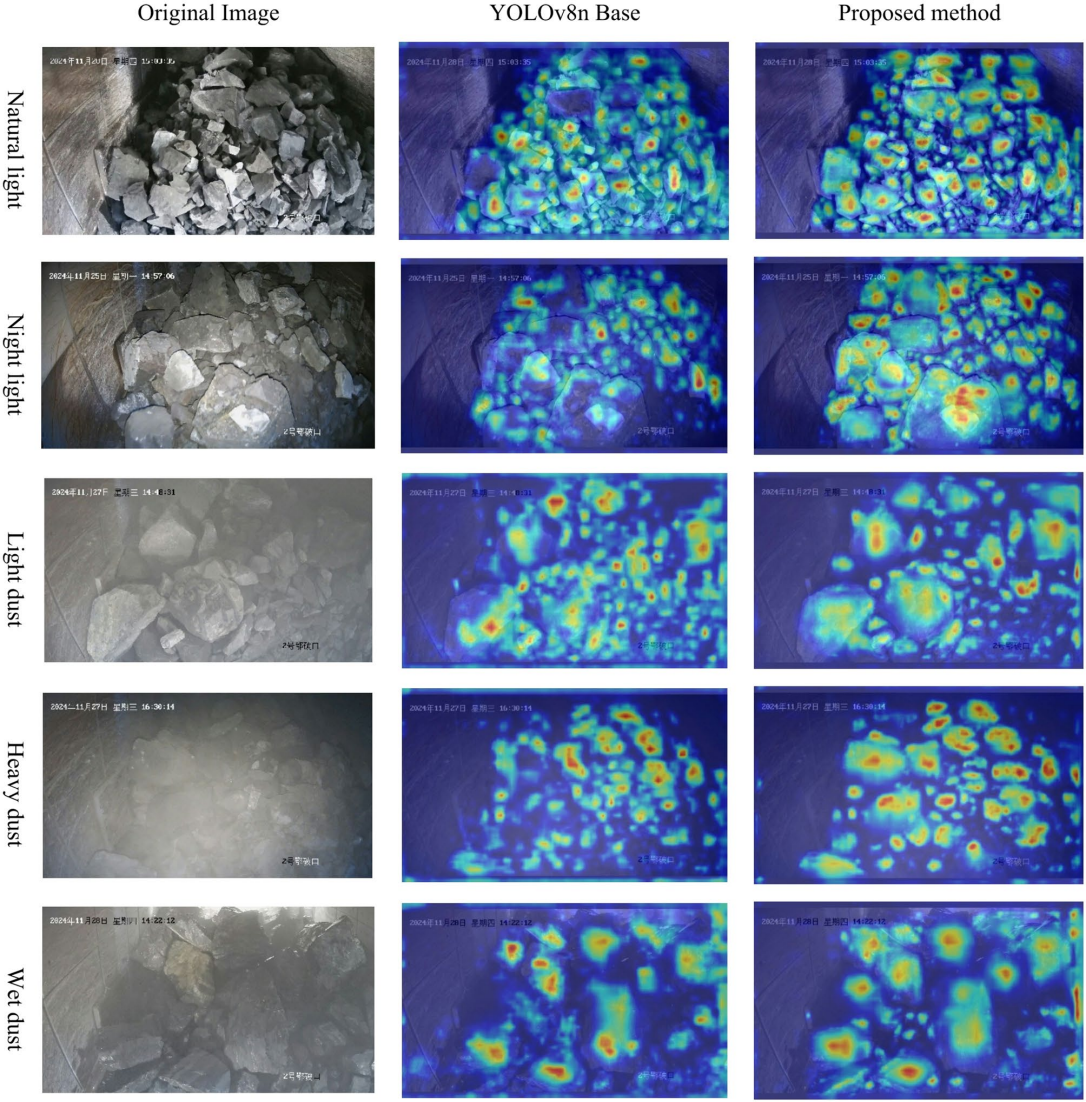
Comparison of heatmap. The top-left and bottom-right corners of each image are the time and location watermarks, which do not affect the model training and detection.

The thermal map was used to further intuitively demonstrate the model’s attention and focus on the blasting ore target in the image. As shown in Fig. 8, for the detection of blasting ore images under natural light and night light, the YOLOv8n benchmark model shows ores and regions with low attention, which leads to missing and false detection in the ore detection results. The improved model focuses on a more comprehensive and uniform area, with almost no missing ores and regions. However, the improved model has more complete and deeper attention than the benchmark model to the single ore position in the image of the ore that has been wet and is in a light and heavy dust environment. This shows that the improved model has a better ability to identify and resolve the ore under the disturbance of dust and wet.

### Conclusions and prospect

Aiming at the blasting ore target detection task in the conveyor belt scene of the feed port of the jaw crusher, this paper proposes a blasting ore particle size detection algorithm based on the integration of an efficient dust removal network and multi-dimensional features. To solve the problems caused by dense ore accumulation, uneven distribution of ore size, drastic change of particle size, different concentrations of dust, scene light change, and ore dry and wet change, such as difficult detection and easy missing and misdetecting, the model was designed and improved. An efficient dust-removal backbone network combining feature attention and composite scaling backbone was constructed. A neck feature fusion network combining convolution and Vmamba sequence model and BiFPN cross-path bidirectional feature fusion architecture was introduced. The multi-dimensional feature fusion capability of Dynamic Head was introduced to optimize the target detection head. A dataset of jaw crusher blasting ore detection, which includes various environment and ore conditions and is classified according to ore particle size, was constructed and tested on this dataset. The experimental results show that compared with the YOLOv8n algorithm, the average accuracy $\overline {P}$ of the proposed algorithm for the detection of ores of eight size categories was increased by 7% and mAP50 by 7.6%. For the fine ore below D5, the detection accuracy $\overline {P}$ increased by 18.8%, and the recall rate $\overline {R}$ increased by 13.8%. While $\overline {P}$ and mAP50 are superior to other mainstream algorithms, the inference speed is significantly higher than other mainstream algorithms, and compared with the YOLOv8n algorithm, a good balance between detection performance and inference speed is achieved.

Our algorithm’s improvement in the accuracy of ore particle size detection helps to more precisely measure the blasting effect of the ore and provides more accurate data for the subsequent research on the relationship between ore particle size and crushing energy consumption. This, in turn, facilitates the quantification of blasting results, the improvement of blasting scheme design, the enhancement of mining energy efficiency, and provides support for intelligent decision-making in open-pit mining operations. Our algorithm has certain robustness against common disturbances in mining, such as dust, changes in scene lighting, and changes in ore dryness and wetness. It also has the ability to operate stably in actual production environments. Additionally, our algorithm is built based on the lightweight YOLOv8 architecture, which enables seamless integration with existing industrial vision platforms and edge computing devices, thus having good compatibility.

Regarding the issue of the area range that this method can identify, the clearly defined identification range shown in the example diagram of this paper is mainly limited by the camera’s field of view (FOV), installation height, and image resolution, rather than being determined by the proposed detection method itself. In fact, the proposed method does not set an inherent upper limit for the identifiable area. The maximum detectable area is in a linear proportion to the camera’s coverage range, and it can be expanded in the following way:

1. Increase the installation height of the camera or use a wide-angle lens;
2. Use a camera with higher resolution;
3. Deploy multiple cameras to achieve overlapping or stitched views.

In our experimental setup, the camera is installed above the feed inlet of the jaw crusher, covering an effective area of approximately 1.75 m by 1.2 m, which is sufficient to capture the entire video stream of the conveyor belt transporting the ores under actual production conditions. Within this range, the proposed method maintains stable detection performance for both fine-grained and large-sized ores, including situations of dense accumulation. Moreover, since the proposed framework processes images in a scale-consistent manner, it can be easily applied to larger scenarios without modifying the architecture. For extremely large-scale scenarios, such as wide conveyor belts or open-pit quarries, this method can be combined with image stitching or sliding window reasoning to achieve an arbitrarily large spatial coverage while maintaining detection accuracy.

Although the ore size detection method based on efficient dust removal network and multi-dimensional feature fusion proposed in this paper achieved good detection performance in complex scenes, there are still some limitations. Although mAE-based physical size evaluation is introduced, the system still relies on bounding boxes rather than accurate contour-level segmentation of irregularly shaped particles. Segmentation-based modules (e.g., Mask2Former and Polygon-RCNN) could be integrated in the future to achieve more refined estimation of particle shape and size. The proposed method is based on RGB images, for ores with large height changes, projection distortion may lead to underestimation of physical size, and only visible ores on the surface can be detected. When ores are accumulated on the transmission belt, the surface ores may block the internal ores. Future research will explore granularity detection methods based on 3D vision, such as jointly training the network with RGB images and depth maps to improve the recognition ability of target thickness and occlusion areas. A multi-camera can be used to realize SfM/MVS modeling and reduce the complete 3D shape of the ore pile.

## Data Availability

The data presented in this study are available on request to the authors and will not be made public due to privacy concerns.

## References

[1] Wang, Q., Li, Z., He, A. & Jian, M. Overview of particle size measurement methods. *J. Jiangsu Inst. Educ. (Nat Sci. Ed.* **24**(02), 25–28 (2007).

[2] Firla, M., Lipnicki, P. & Lewandowski, D. Image processing algorithm for the assessment of the ore fragmentation size distribution. In *2019 24th IEEE International Conference on Emerging Technologies and Factory Automation (ETFA)* 505–512 (2019). 10.1109/ETFA.2019.8869462

[3] Yang, Z., Ding, H., Guo, L. & Lian, M. Superpixel image segmentation-based particle size distribution analysis of fragmented rock. *IEEE Access.* **9**, 59048–59058. 10.1109/ACCESS.2021.3072998 (2021).

[4] Luo, X., Lin, L. & Cai, G. Online detection system for ore particle size based on image processing. *Instrum. Technol. Sens.* **80**(7), 63–64 (2015).

[5] Ma, L., Zhang, Y., Song, G., Ma, Z. & Lu, T. Ore granularity detection and analysis system based on image processing. In *2019 Chinese Control and Decision Conference (CCDC)* 359–366. 10.1109/CCDC.2019.8832862 (2019).

[6] Zhang, J., Feng, X. & Zhang, J. Application of improved wavelet and watershed algorithm in ore particle size detection. *Mech. Des. Manuf. No*. **6**, 290–294. 10.19356/j.cnki.1001-3997.2022.06.039 (2022).

[7] Liu, X., Zhang, Y., Jing, H., Wang, L. & Zhao, S. Ore image segmentation method using U-net and Res_Unet convolutional networks. *RSC Adv.* **10**(16), 9396–9406. 10.1039/C9RA05877J (2020). 35497237 10.1039/c9ra05877j PMC9050132

[8] Gu, Q., Wei, F., Guo, M., Jiang, S. & Ruan, S. Crushed ore image segmentation method based on improved HED network model. *Laser Optoelectron. Prog*. **59**(2), 262–270 (2022).

[9] Li, F., Liu, X., Yin, Y. & Li, Z. DDR-Unet: A high-accuracy and efficient ore image segmentation method. *IEEE Trans. Instrum. Meas.* **72**, 1–20. 10.1109/tim.2023.3317480 (2023).37323850

[10] Bo, J., Zhang, C., Fan, C. & Li, H. Improved YOLOv3 method for ore conveyor belt debris detection. *Comput. Eng. Appl.* **57**(21), 248–255 (2021).

[11] Sandler, M., Howard, A., Zhu, M., Zhmoginov, A. & Chen, L. C. Mobilenetv2: Inverted residuals and linear bottlenecks. In *Proceedings of the IEEE Conference on Computer Vision and Pattern Recognition* 4510–4520 (2018). 10.1109/cvpr.2018.00474

[12] Gao, X., Li, B., Wang, L., Li, L. & Wang, X. Coal gangue recognition based on object detection network. *Chin. J. Powder Technol.* **27**(4), 77–83. 10.13732/j.issn.1008-5548.2021.04.010 (2021).

[13] Lin, T. Y. et al. Feature pyramid networks for object detection. In *2017 IEEE Conference on Computer Vision and Pattern Recognition (CVPR)* 936–944 (CVPR, 2017). 10.1109/CVPR.2017.106

[14] Xie, T.* Research on Fine-Grained Mineral Recognition Based on Deep Learning*. Master’s Thesis, China University of Mining and Technology, Xuzhou, China 10.27623/d.cnki.gzkyu.2020.001335 (2021).

[15] Xie, B. *Design of an Ore Particle Size Recognition System Based on Convolutional Neural Network*. Master’s Thesis, Northeastern University, Shenyang, China. 10.27007/d.cnki.gdbeu.2022.000537 (2025).

[16] Qin, X., Wang, Z., Bai, Y., Xie, X. & Jia, H. FFA-net: Feature fusion attention network for single image dehazing. In *Proceedings of the AAAI Conference on Artificial Intelligence* 07, Vol. 34, no. 07 10.1609/aaai.v34i07.6865 (2020).

[17] Tan, M. & Le, Q. EfficientNet: Rethinking model scaling for convolutional neural networks. In *Proceedings of the 36th International Conference on Machine Learning, Proceedings of Machine Learning Research* (eds Chaudhuri, K. & Salakhutdinov, R.) 6105–6114, Vol. 97 (PMLR, 2019). [Online]. Available: https://proceedings.mlr.press/v97/tan19a.html.

[18] Liu, Y. et al. VMamba: visual state space model. In *Advances in Neural Information Processing Systems*, (eds Globerson, A., Mackey, L., Belgrave, D., Fan, A., Paquet, U., Tomczak, J. & Zhang, C.) 103031–103063 (Curran Associates, Inc., 2024). 10.48550/arXiv.2401.10166. (2024).

[19] Tan, M., Pang, R. & Le, Q. V. EfficientDet: Scalable and efficient object detection. In *2020 IEEE/CVF Conference on Computer Vision and Pattern Recognition (CVPR)* 10778–10787 10.1109/CVPR42600.2020.01079 (2020).

[20] Dai, X. et al. June., Dynamic head: Unifying object detection heads with attentions. In *2021 IEEE/CVF Conference on Computer Vision and Pattern Recognition (CVPR)* 7369–7378 (Nashville, TN, USA: IEEE, 2021). 10.1109/CVPR46437.2021.00729.

[21] Hayat, M. Squeeze & excitation joint with combined channel and Spatial attention for pathology image super-resolution. *Frankl. Open.* **8**, 100170. 10.1016/j.fraope.2024.100170 (2024).

[22] Peng, S., Zhang, X., Zhou, L. & Wang, P. YOLO-CBD: Classroom behavior detection method based on behavior feature extraction and aggregation. Sensors. **25**(10), 3073. 10.3390/s25103073 (2025). 10.3390/s25103073 PMC12115732 40431867

[23] Wang, P., Hu, Y., Peng, S. & Zhou, L. EMANet: An ancient text detection method based on enhanced-EfficientNet and multidimensional scale fusion. *IEEE Internet Things J.* **11**(19), 32105–32116. 10.1109/JIOT.2024.3423667 (2024).

[24] Gu, A. & Dao, T. *Mamba: Linear-Time Sequence Modeling with Selective State Spaces*. arXiv. 10.48550/ARXIV.2312.00752 (2023).

[25] Zhang, S., Chi, C., Yao, Y., Lei, Z. & Li, S. Z. Bridging the gap between anchor-based and anchor-free detection via adaptive training sample selection. In *2020 IEEE/CVF Conference on Computer Vision and Pattern Recognition (CVPR)* 9756–9765. (Seattle, WA, USA: IEEE, 2020). 10.1109/CVPR42600.2020.00978.

[26] Zhu, B. et al. *AutoAssign: Differentiable Label Assignment for Dense Object Detection.* arXiv: arXiv:2007.03496. 10.48550/arXiv.2007.03496 (2020).

[27] Ren, S., He, K., Girshick, R., Sun, J., Faster, R-C-N-N. & Towards real-time object detection with region proposal networks. *IEEE Trans. Pattern Anal. Mach. Intell.* **39**(6), 1137–1149. 10.1109/TPAMI.2016.2577031 (2017). 27295650 10.1109/TPAMI.2016.2577031

[28] Zheng, Z. Localization distillation for dense object detection. In *Proceedings of the IEEE/CVF Conference on Computer Vision and Pattern Recognition (CVPR)* 9407–9416. 10.48550/arXiv.2102.12252 (2022).

[29] Kong, T. et al. FoveaBox: Beyound anchor-based object detection. *IEEE Trans. Image Process.* **29**, 7389–7398. 10.1109/TIP.2020.3002345 (2020).

[30] Feng, C., Zhong, Y., Gao, Y., Scott, M. R. & Huang, W. TOOD: Task-aligned one-stage object detection. In *2021 IEEE/CVF International Conference on Computer Vision (ICCV)* 3490–3499 10.1109/ICCV48922.2021.00349 (2021).

[31] Tian, Z., Shen, C., Chen, H. & He, T. FCOS: Fully convolutional one-stage object detection. In *2019 IEEE/CVF International Conference on Computer Vision (ICCV)* 9626–9635 10.1109/ICCV.2019.00972 (2019).

[32] Li, X. et al. Generalized focal loss: learning qualified and distributed bounding boxes for dense object detection. In *Advances in Neural Information Processing Systems* 21002–21012, (Curran Associates, Inc., 2020). 10.48550/arXiv.2006.04388.

[33] Wang, A. et al. *YOLOv10: Real-Time End-to-End Object Detection*. arXiv: arXiv:2405.14458. 10.48550/arXiv.2405.14458 (2024).

[34] Alif, M. A. R. & Hussain, M. *YOLOv12: A Breakdown of the Key Architectural Features*. arXiv:arXiv:2502.14740. 10.48550/arXiv.2502.14740 (2025).

